# Sequence-specific cleavage of dsRNA by Mini-III RNase

**DOI:** 10.1093/nar/gkv009

**Published:** 2015-01-29

**Authors:** Dawid Głów, Dariusz Pianka, Agata A. Sulej, Łukasz P. Kozłowski, Justyna Czarnecka, Grzegorz Chojnowski, Krzysztof J. Skowronek, Janusz M. Bujnicki

**Affiliations:** Laboratory of Bioinformatics and Protein Engineering, International Institute of Molecular and Cell Biology in Warsaw, ul. Ks. Trojdena 4, 02-109 Warsaw, Poland

## Abstract

Ribonucleases (RNases) play a critical role in RNA processing and degradation by hydrolyzing phosphodiester bonds (exo- or endonucleolytically). Many RNases that cut RNA internally exhibit substrate specificity, but their target sites are usually limited to one or a few specific nucleotides in single-stranded RNA and often in a context of a particular three-dimensional structure of the substrate. Thus far, no RNase counterparts of restriction enzymes have been identified which could cleave double-stranded RNA (dsRNA) in a sequence-specific manner. Here, we present evidence for a sequence-dependent cleavage of long dsRNA by RNase Mini-III from *Bacillus subtilis* (BsMiniIII). Analysis of the sites cleaved by this enzyme in limited digest of bacteriophage Φ6 dsRNA led to the identification of a consensus target sequence. We defined nucleotide residues within the preferred cleavage site that affected the efficiency of the cleavage and were essential for the discrimination of cleavable versus non-cleavable dsRNA sequences. We have also determined that the loop α5b-α6, a distinctive structural element in Mini-III RNases, is crucial for the specific cleavage, but not for dsRNA binding. Our results suggest that BsMiniIII may serve as a prototype of a sequence-specific dsRNase that could possibly be used for targeted cleavage of dsRNA.

## INTRODUCTION

Ribonucleases (RNases) play a critical role in RNA processing and degradation by participation in a variety of biochemical reactions that involve exo- and endonucleolytic cleavage of RNA molecules. Exoribonucleases (exoRNases) degrade RNAs starting at their termini in a sequence-independent manner, whereas endoribonucleases (endoRNases) cleave internally single- or double-stranded RNA (dsRNA) molecules. Many endoRNases exhibit substrate specificity, but their target site is usually limited to one or a few specific nucleotides in single-stranded RNA and often in a context of a particular three-dimensional structure of the substrate. Examples include endoRNase T1 that cleaves single-stranded RNA with high specificity at guanosine residues (1), endoRNase RegB that targets its mRNA substrates with an almost absolute specificity in the middle of the tetranucleotide GGAG (2) and α-sarcin that cleaves 28S rRNA in the GAGA sequence (3). In Eukaryota, several RNases have been described; however, none of them exhibit sequence specificity (4). There were numerous attempts to engineer the specificity of single-stranded RNases i.e. a fusion of an RNase T1 and the TAT peptide (5), fusion of a PIN nuclease with a PUF domain (6), but no similar efforts were reported for RNases that act on dsRNA.

RNase III is an archetypal endoRNase that cleaves dsRNA. It shares an evolutionarily conserved catalytic domain with a large group of enzymes in the RNase III superfamily. The classification of RNase III enzymes depends on the presence of various functional elements in addition to the catalytic domain (7). These elements enable substrate selection and binding, regulation of activity, and interaction with other proteins. Class 1, i.e. orthodox RNase III enzymes, are homodimeric, each identical subunit contains a dsRNA binding domain (dsRBD) and a single RNase III domain. Class 2 and class 3 enzymes are represented by Drosha and Dicer respectively, which comprise two RNase III domains along with a single dsRBD. In addition, enzymes belonging to the class 2 possess a polyproline domain and those in the class 3 usually have three additional domains: e.g. DExD helicase, DUF283 and PAZ. The class 2 and class 3 enzymes typically act as monomeric species. The class 4 enzymes, called Mini-III, are homodimeric enzymes and consist solely of the RNase III domains.

The biochemical properties of the archetypal RNase III from *Escherichia coli* were extensively studied. It was postulated that the efficiency of cleavage is profoundly affected by the structural features of the substrate that are close to the cleavage site (8). The weak consensus sequence around the RNase III cleavage site (WNAGWGNNCWUNNN^∧^NAWGNNCWCUNW, where W stands for A or U and N stands for any residue, and ^∧^ indicates the scissile phosphodiester bond) was derived from the comparison of sequences from the previously reported substrates (9). This data set included not only regular dsRNAs but also molecules that contained mismatches and bulges. Another study defined the sequence antideterminants of RNase III activity. The presence of specific Watson–Crick (W-C) base-paired sequences in two discrete segments of the substrate dsRNA was found to strongly inhibit cleavage. The two segments, termed proximal and distal boxes, were identified through alignment of 10 well-characterized RNase III substrates. The proximal box located at positions -4, -5 and -6 relative to the cleavage site was found to lack the W-C bp GC/CG in the middle position and to have GC under-represented on the flanks. The distal box located at positions -11 and -12 relative to the cleavage site was found to lack the W-C bp UA in the first position and GC in the second (10).

In the aforementioned studies, sequence preference of RNase III relates to the nucleotides in the vicinity of the cleavage site, but not to those directly next to the scissile phosphodiester bond. In this enzyme, the dsRBD, a domain that facilitates substrate binding, acts as an anchor for the nuclease domain. Initially, the dsRBD was thought to recognize exclusively the shape of the A-form of the RNA helix. However, the appearance of a number of dsRBDs structures and proteins that have a dsRBD in complex with substrates provided a more comprehensive insight into the molecular basis of dsRNA recognition and a potential explanation for the observed cleavage preference of such enzymes [reviewed in (11)]. Structural analysis of the first structure of an RNase III-RNA complex with the substrate revealed that it contacts dsRNA through four regions. They are termed the RNA binding motifs (RBMs), two of which are present in the catalytic domain and two are in the dsRBD (12). The substrate specificity of RNase III is achieved mainly by the interaction of dsRBD with the O2′ hydroxyls and recognition of the width of the dsRNA grooves. Additionally, the dsRBD makes one specific contact between a glutamine and a guanine residue in the double-stranded region of the RNA. Other examples of base-specific contacts were observed, enabling the identification of two residues, methionine and glutamine, within the two regions in the dsRBD that interact with the adenine and guanine residues, respectively (13,14). The dsRBDs were also found to recognize such structures as apical hairpin loops in RNA (15,16).

In contrast to other classes of RNase III enzymes, members of class 4 (the Mini RNase III family) do not possess any additional domains. Since they do not contain a dsRBD, the mechanism by which they recognize the substrate was postulated to be different from the other RNase III superfamily members (17). There are also minor differences in the structure of the catalytic domain between Mini-III RNases and the canonical RNase III proteins from class 1 (Supplementary Figure S1). A long loop between helices α5 and α6 of the canonical RNases III, including the RBM 4, in Mini-III RNases is replaced by an additional helix, called α5b and a short loop connecting the α5b and α6 helices (called loop α5b-α6). An enzyme from *Bacillus subtilis* (BsMiniIII) was shown to be involved in 23S rRNA maturation. The natural substrate for BsMiniIII is 23S pre-rRNA, in which the 3′ and 5′ ends of the molecule are removed to yield a mature 23S rRNA (17). The cleavage site of BsMiniIII is located at the end of the stem formed by 3′ and 5′ ends of the mature 23S rRNA, close to a three-way junction formed by the substrate. BsMiniIII *in vitro* activity was shown to be stimulated by the ribosomal protein L3 bound to the 3′ end of the 23S rRNA. There is indirect evidence that the protein L3 enhances the cleavage of the substrate by changing the conformation of the RNA (18).

Here, we present evidence that BsMiniIII is capable of cleaving a long dsRNA substrate in a sequence-specific manner. To our knowledge, this is the first report of a sequence-specific recognition and cleavage of dsRNA by an RNase.

## MATERIALS AND METHODS

### Gene cloning, protein expression and purification

The open reading frame of the *yazC* gene from *B. subtilis* ATCC 23857 strain was amplified using the primers BSUf/BSUr (Supplementary Table S1). The polymerase chain reaction (PCR) product was inserted between the NdeI and XhoI sites of the pET30b vector (Novagen) resulting in a plasmid pETBsu that encoded BsMiniIII endoRNases with a C-terminal His_6_-tag. Variants of the pETBsu encoding BsMiniIII with either deletions of parts of the α5b-α6 loop, pETBsuΔ10 (deletion of residues K86-D95) and pETBsuΔ5 (deletion of residues P91-D95) or single amino acid substitutions K86N, K92H, K92R, K92D, K92E, N93H, N93R, N93D, N93E, D95H, D95R, D95K, D95E were generated by PCR (pairs of primers indicated in Supplementary Table S1). All constructs were verified by DNA sequencing. Plasmids were transformed into *E. coli* BL21 (DE3) strain. The bacterial cultures were grown to mid-log phase. Protein expression was induced by addition of isopropyl β-D-1-thiogalactopyranoside (IPTG) to 1 mM, followed by 3 h of bacterial growth at 37°C. The cell pellet was resuspended in L0 buffer (50 mM sodium phosphate pH 8.0, 300 mM NaCl, 10 mM imidazole, 10% glycerol, 10 mM 2-mercaptoethanol, 1 mM phenylmethylsulfonyl fluoride (PMSF)). The cells were lysed using a cell disruptor (Constant Systems Ltd.) at 20 kpsi. The lysate was cleared by centrifugation, 40 000 g for 30 min at 4°C. The proteins were purified by batch Ni-NTA affinity chromatography on a His-Select Nickel Affinity Gel (Sigma). The clarified lysate was incubated with the resin for 1 h at 4°C with constant shaking. Afterward, two washing steps were performed with L1 buffer (L0 buffer supplemented with 2 M NaCl) and with L2 buffer (L0 buffer supplemented with 20 mM imidazole). The His-tagged proteins were eluted with E buffer (L0 supplemented with 250 mM imidazole).

For crystallization trials, the E105Q mutant of BsMiniIII with the C-terminal His_6_-tag was expressed in the *E. coli* BL21 (DE3) strain. Protein expression and purification was conducted as previously with the exception of the elution step which was performed with L0 buffer containing 60 mM imidazole. The enzyme containing fractions were pooled together and the buffer was exchanged using diafiltration to B buffer (50 mM Tris-HCl pH 8.0, 300 mM NaCl, 10% glycerol, 1 mM dithiothreitol (DTT)) and samples were applied to a HiLoad 16/60 Superdex 200 size exclusion column (GE Healthcare). The fractions containing the purified protein were pooled and concentrated by ultrafiltration (Vivaspin, Millipore).

The protein concentration was measured on a NanoDrop 1000 spectrophotometer. The homogeneity of the protein samples was determined by 15% sodium dodecyl sulphate-polyacrylamide gel electrophoresis (SDS-PAGE) gel and assessed to be higher than 95%. The purity of the wt enzyme preparation was also confirmed by mass spectrometry (Supplementary Figure S2 and Supplementary Table S2).

### RNA substrate preparation

The dsRNA Φ6 bacteriophage genome consisting of three linear segments (L-6374 bp, M-4063 bp and S-2948 bp) was purchased from Finnzymes. We have also used a set of shorter, dsRNA substrates produced by enzymatic *in vitro* synthesis.

To generate a template for the pKS-ACCU dsRNA synthesis, we have modified the pBluescript II KS(+) vector (Stratagene) by deleting the 45 bp sequence between the T7 promoter and the BamHI site (C646-G690), by a PCR reaction with primers KSf and KSr (Supplementary Table S1), followed by a subsequent circularization, resulting in a vector pKSRNA. Subsequently, the double-stranded oligonucleotide NC1/NC2 was inserted into a SmaI site of the pKSRNA plasmid generating the pKS-ACCU plasmid. The PCR products generated by amplification of the 250 bp region in the pKS-ACCU with NC3 and NC4 primers (Supplementary Table S1), served as templates for 234 bp dsRNAs synthesis using a Replicator RNAi kit (Thermo Scientific). In order to obtain particular shorter dsRNA fragments of the Φ6 genome, in the first step single-stranded DNA was generated by reverse transcription using Maxima Reverse Transcriptase (Thermo Scientific) and random hexamers on the Φ6 genomic RNA template. In the second step, specific oligonucleotides and the obtained single-stranded DNA were used in the PCR reaction (Supplementary Table S1) to ampify the desired region. One oligonucleotide of each pair contained T7 RNA Polymerase promoter's sequence while the second one contained Φ6 RNA Replicase promoter's sequence. The final PCR products served as templates for dsRNA synthesis using Replicator RNAi kit (Thermo Scientific).

Saturation mutagenesis of each position in the cleavage site sequence in pKS-ACCU dsRNA was done by a PCR reaction with the primer sets Sub83–Sub96 (Supplementary Table S3) and the pKS-ACCU plasmid as a template. The PCR products were circularized and sequenced to select constructs with the desired substitutions.

The 30 bp dsRNA substrate was prepared by annealing RNA oligonucleotides 30f and 30r (Supplementary Table S1). The 5′ end of one strand was labeled with [γ-33P] ATP using a T4 polynucleotide kinase and annealed with the unlabeled complementary oligonucleotide.

### dsRNA cleavage assays

All cleavage reactions were carried out at 37°C. The reaction buffer for Mini-III contained 10 mM Tris-HCl pH 7.5, 5 mM NaCl, 1 mM MgCl_2_, 100-μg/ml bovine serum albumin (BSA). The precise cleavage conditions are stated under figures. Cleavage reactions were terminated by the addition of gel loading buffer containing ethylenediaminetetraacetic acid (10 mM final concentration) and 1/10 volume of phenol:chloroform. The cleavage products were separated by agarose or native polyacrylamide gel electrophoresis and visualized by UV light after staining with ethidium bromide using a LAS4010 imaging system (GE Healthcare). The cleavage efficiencies of the dsRNAs substrates at each time point were calculated from densitometric analysis of electropherograms with ImageQuantTL software. Cleavage rates were measured by linear regression of the cleavage efficiency versus time plots and then normalized for the amount of enzyme. Cleavage rates of substitution variants were normalized to the cleavage rate of wt BsMiniIII, which was set to a value of 1. The radiolabeled samples were exposed on Storage Phosphor Screen (GE Healthcare) for 2 h. The autoradiographs were visualized on a Storm scanner (GE Healthcare).

### Primer extension with reverse transcriptase

Cleavage products of pKS-ACCU dsRNA were purified by isolation from the native 6% polyacrylamide gel in a Tris/borate/ethylenediaminetetraacetic acid (TBE) buffer using a crush and soak elution method (19) with 2 M ammonium acetate pH 5.3 followed by ethanol precipitation. One picomole of 5′ (NC5, NC6) primers radiolabeled with [γ-33P] ATP using a T4 polynucleotide kinase (Supplementary Table S1) was annealed to the dsRNA products. Reactions were carried out using Avian Myeloblastosis Vi­rus (AMV) reverse transcriptase (Thermo Scientific) according to the manufacturer's protocol. Products were analyzed by 6% TBE polyacrylamide/urea gel electrophoresis followed by autoradiography.

### RNA sequencing analyses

The product of the time-limited cleavage (after 10 and 15 min) of 5 μg of the Φ6 genome with BsMiniIII was denatured for 1 min at 95°C and cooled quickly on ice. Then 50 pmoles of 5′ preadenylated adapters (UniShPreA; Supplementary Table S1) were ligated to 3′ ends of the cleavage products with truncated KQ T4 RNA Ligase 2 (New England Biolabs) for 16 h at 16°C. The unligated adapters were removed on GeneJET RNA Cleanup and Concentration Micro columns (Thermo Scientific) and purified RNA was used as a template for the reverse transcription reaction with Maxima reverse transcriptase (Thermo Scientific) and the primer UniShRT complementary to the adapter UniShPreA. The reactions were incubated for 5 min at 50°C and terminated by heating for 5 min at 80°C. The cDNA was purified with a GeneJET DNA Cleanup Micro Kit (Thermo Scientific) and the second preadenylated adaptor PreA3Univ was ligated to the cDNA 3′ ends by Thermostable 5′ App DNA/RNA Ligase (New England Biolabs). After removal of unligated adapters with GeneJET DNA Cleanup Micro Kit, the cDNA was amplified in a PCR reaction (15–18 cycles of amplification were used). The PCR products were resolved in the agarose gel electrophoresis where fraction of size between 200 and 700 bp was selected, purified from agarose gel slices with GeneJET Gel Extraction Kit (Thermo Scientific) and subjected to Next-Generation Sequencing on MiSeq (Illumina) platform at Genomed.

Reads obtained from MiSeq (Illumina) were mapped to the Φ6 genome using Bowtie 2 (version 0.2) (20) available on the Galaxy platform (http://usegalaxy.org (21)) in end-to-end mode and default parameters. Two distinct approaches were used to analyze the high-throughput sequencing. The first approach used the top 200 reads (the most frequent, unique reads in the pool) of 8 nucleotides each. The motifs were generated by a *de novo* motif discovery tool MEME (22) with default parameters except the setting for minimum width of the motifs which was changed from 6 to 8. In the second approach, we used all reads, taking into account their frequency (due to the limitation of maximal input sequence number in MEME, this time DREME (23) was used). As an input, the program takes both a positive sequence set (up to 14 nucleotides sequences centered on the cleavage site) and a negative set (background sequences generated from the Φ6 genome using a 14-nucleotide sliding window). The cleavage sites detected on both strands were summed and then DREME was allowed to search the positive strand and its reverse complement to find statistically over-represented motifs. To fix the motif search to its central part representing the cleavage site, the DREME program was run incrementally with increasing sequence length (from 4 to 14 nucleotides). Then, the longest motif was chosen which does not cause a dramatic drop in both submotifs diversity and motif presence in the positive set. Keeping in the mind these two conditions, the optimal motif of 6 nucleotides was obtained.

### Crystallization

Freshly purified protein, 6.5 mg ml^−1^ in 50 mM Tris-HCl pH 8.0, 300 mM NaCl, 10% glycerol, 1 mM DTT, was used for crystallization trials using Hampton Research Screens. The crystal growth occurred at 20°C over a period of ∼30 days. A diffraction-quality crystal was obtained using Crystal Screen I at condition No. 22, consisting of 0.2 M sodium acetate trihydrate; 0.1 M Tris hydrochloride pH 8.5; 30% w/v polyethylene glycol 4000.

### Crystal structure determination

The C 2 2 21 crystal form diffracted up to 1.8 Å on beamline 14.1 at BESSY (Berliner Elektronenspeicherring-Gesellschaft für Synchrotronstrahlung, Berlin, Germany). The diffraction data were integrated with X-ray Detector Software (XDS) (24). Crystals contained one monomer of BsMiniIII in the asymmetric unit. The structure was solved by molecular replacement with the program PHASER (25) using the *Bacillus cereus* MiniIII structure (PDB code 1u61) as the search model. The crystal structure model was corrected manually in Coot (26) and refined using REFMAC 5.8 (27) (Supplementary Table S4). The crystal structure model was deposited in PDB with the accession code 4OUN.

## RESULTS

### Cleavage of dsRNA with BsMiniIII RNase results in a distinct banding pattern

**Figure 1.**
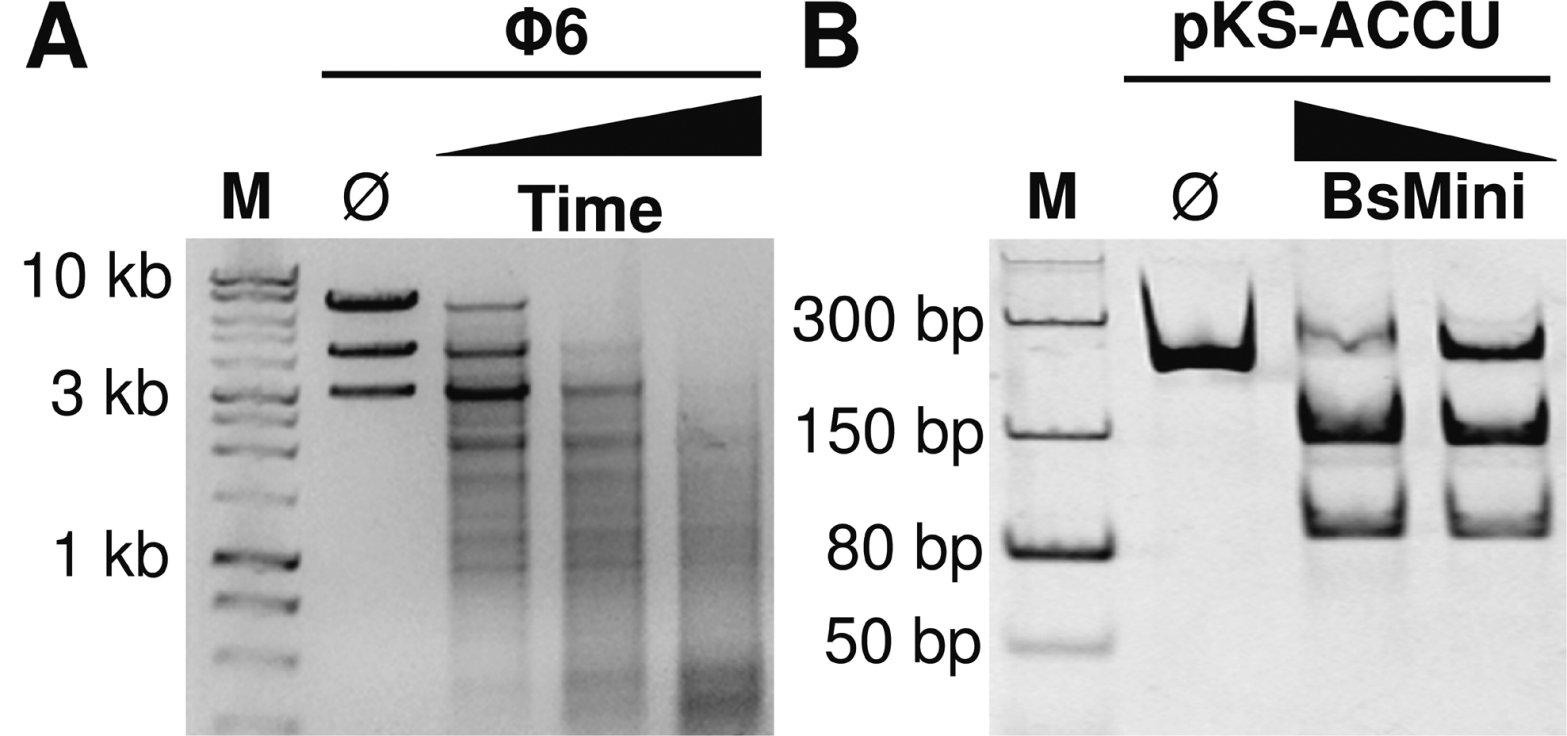
Activity of BsMiniIII. (**A**) Cleavage of the Φ6 dsRNA substrate. Reactions aliquots were taken at 15, 30 and 45 min at 37°C. Each 10 μl aliquot contained 3.1 μg of wt BsMiniIII (8.3 μM) and 500 ng of Φ6 dsRNA. Samples were resolved on an agarose gel. The Ø indicates an untreated substrate. (**B**) Cleavage of the pKS-ACCU dsRNA substrate by BsMiniIII. Reactions were carried out for 1 h at 37°C with 125 nM BsMiniIII and 50 nM substrate. Samples were resolved on a native polyacrylamide gel. M: dsDNA molecular weight marker. The Ø indicates an untreated substrate. The nucleic acids were visualized after staining with ethidium bromide.

Specific endoRNase activity of BsMiniIII was analyzed using the Φ6 bacteriophage dsRNA as a substrate. The conditions for specific dsRNA cleavage for BsMiniIII were: Tris-HCl pH 7.5, 5 mM NaCl, 1 mM MgCl_2_, 0.1 mg/ml BSA. Following a time-limited cleavage of the Φ6 dsRNA in the optimal conditions, a pattern of specific bands was observed, suggesting that the enzyme acts as a dsRNase and preferentially cleaves certain sequences present in the substrate (Figure 1A). Replacement of Mg^2+^ ions with Ca^2+^ led to complete inhibition of cleavage, whereas the replacement with Mn^2+^ caused extensive degradation of the substrate without the appearance of discrete bands (data not shown). The nucleolytic activity of the canonical RNase III was also shown to be dependent on the type of divalent ion (28). This feature of RNase BsMiniIII also to some extent resembles the properties of many Type II restriction enzymes that act on dsDNA (29).

### Characterization of BsMiniIII sequence preference

In order to characterize the preferred target sites of BsMiniIII, we cloned five dsDNA oligonucleotides with sequences corresponding to the fragments of segment L of the Φ6 genome in the pKSRNA vector. The fragments were chosen based on a preliminary primer extension analysis of the products of BsMiniIII cleavage of the L segment. The dsRNAs synthesized from these constructs were 234 bp long and each contained a different 30 bp fragment of phage Φ6 flanked by the same sequence derived from the plasmid vector. The patterns of bands resulting from BsMiniIII cleavage indicated that the site cleaved with the highest efficiency in all substrates tested is located in the sequence derived from the vector. Some of the substrates were cleaved only in this site and cleavage resulted in the appearance of two fragments, ∼150 and 90 bp (Figure 1B). Therefore, we chose one of these substrates (pKS-ACCU dsRNA) to precisely define the cleavage site using a primer extension assay.

**Figure 2.**
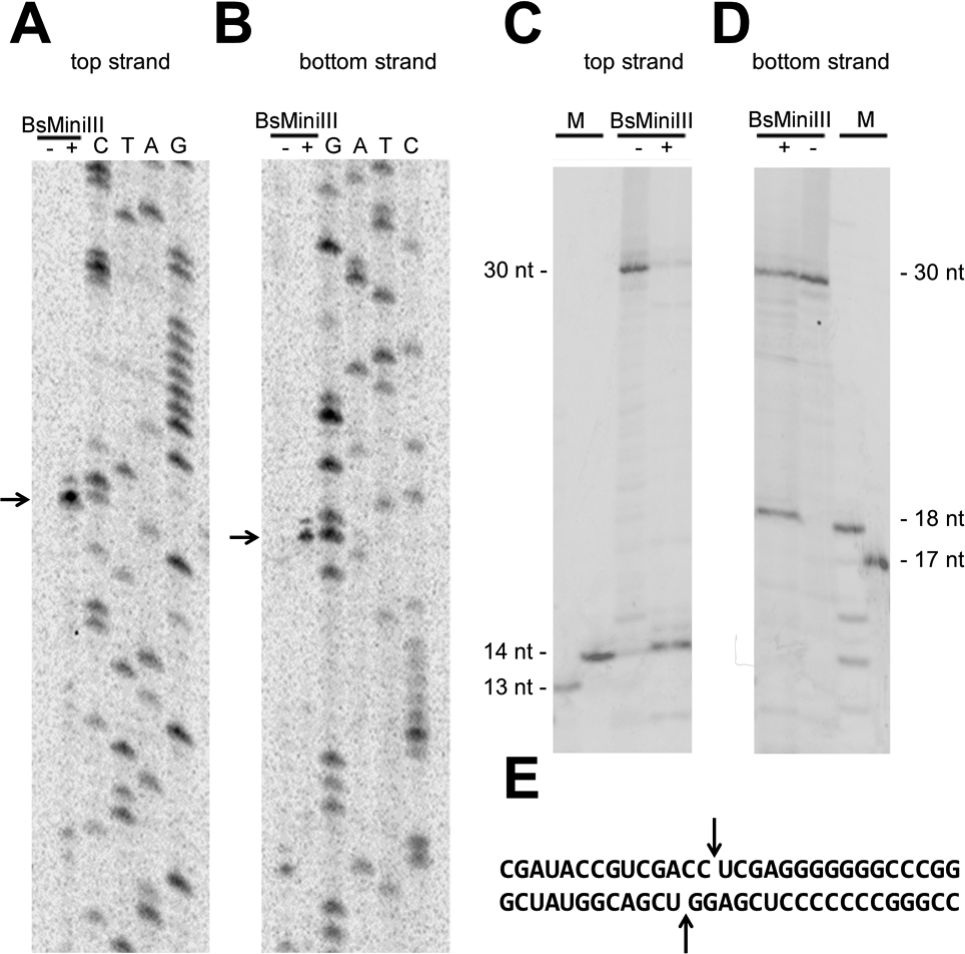
Identification of the BsMiniIII cleavage site in dsRNA. (**A, B**) Mapping of the cleavage site in the pKS-ACCU molecule by primer extension. The detection of the cleavage by primer extension reaction using a ^33^P-labeled primer complementary to the top strand (A) and to the bottom strand (B). The reactions were resolved on a denaturing polyacrylamide gel and visualized using autoradiography. The arrows indicate the strong termination of the primer extension. A dash and a plus sign indicate a primer extension reaction carried out on an uncleaved and a cleaved template, respectively. (**C, D**) Mapping of the cleavage site in a 30 bp dsRNA oligonucleotide (sequence shown in panel (**E**)). Detection of the cleavage of a ^33^P-labeled 30 bp fragment that corresponds to the top strand (C) and to the bottom strand (D) of the pKS-ACCU dsRNA molecule. A dash and a plus sign indicate an uncleaved and cleaved oligonucleotide, respectively. The 13, 14, 17 and 18-nt markers are ^33^P-labeled fragments of the 30-nt oligonucleotide shortened at the 3′ end. (E) The sequence of the dsRNA oligonucleotide substrate. The arrows indicate the cleavage site.

The primer extension analysis, using the top strand of the pKS-ACCU dsRNA as a template, resulted in a strong termination signal at a position corresponding to residue 90 of the top stand of the substrate and a weaker termination signal on the next residue (corresponding to position 89) (Figure 2A). In the primer extension assay of the bottom strand, a strong termination appeared at the position corresponding to residue 89 of the top stand, and a weaker one corresponding to residue 90 (Figure 2B). The last residues observed in the primer extension products (corresponding to the weak termination signals) were likely thymidines added by the reverse transcriptase enzyme in a non-template manner (30). In order to resolve the ambiguity of interpretation of the primer extension experiment results, caused by the potential addition of non-templated residues by the reverse transcriptase, a 5′-end-labeled 30 bp dsRNA with the sequence encompassing the cleavage site was used as a substrate. The dsRNA oligonucleotide was cleaved at the top strand between the 14th and 15th residues (Figure 2C) and at the bottom strand between the 18th and 19th residues, thereby confirming the initial interpretation of the primer extension experiment (Figure 2D). Thus, like the orthodox RNase III, BsMiniIII generates 2-nucleotide 3′ overhangs after cleavage. In addition, we tested its ability to use a single-stranded RNA as a substrate; however, we did not detect any cleavage (Supplementary Figure S3).

The sequence preference of BsMiniIII was studied in detail by systematic substitutions of each of the 14 positions surrounding the cleavage site and corresponding to positions 83–96 in the pKS-ACCU dsRNA substrate. We generated a series of variants, in which every residue from the chosen fragment was subjected to mutagenesis. Altogether, 42 dsRNA variants, each with a single substitution, were tested, and their susceptibilities to cleavage by BsMiniIII were compared to the cleavage susceptibility of the original substrate (summary of results in Supplementary Figure S4). Based on this analysis, we derived the following consensus sequence: (**G**/A/U)(**A**/U)CC^∧^(**U**/A)(**C**/A/U)(**G**/**A**/U), where the most efficiently cleaved residue variants are marked in bold and ^∧^ indicates the scissile phosphodiester bond. According to the results of these assays, the CC dinucleotide between positions 89 and 90 was a strong determinant for BsMiniIII cleavage, as no substitutions of either of these two nucleotides were tolerated in the context of the substrate sequence analyzed at this stage.

In an attempt to characterize BsMiniIII cleavage preference more comprehensively, we used high-throughput sequencing of the ends generated after a 10 min-limited cleavage of the Φ6 genome. We obtained 236 860 reads that were uniquely mapped to the phage Φ6 genome sequence. In the control experiment, when an uncleaved Φ6 genome was subjected to the same analysis, almost 93% of the reads started at one of the six ends of the three genome segments. In comparison, after limited cleavage of the Φ6 genome, only ∼7% started at one of the genome segment ends. Thus, we inferred that the reads, which started within the substrate, resulted primarily from the cleavage of dsRNA by BsMiniIII.

**Figure 3.**
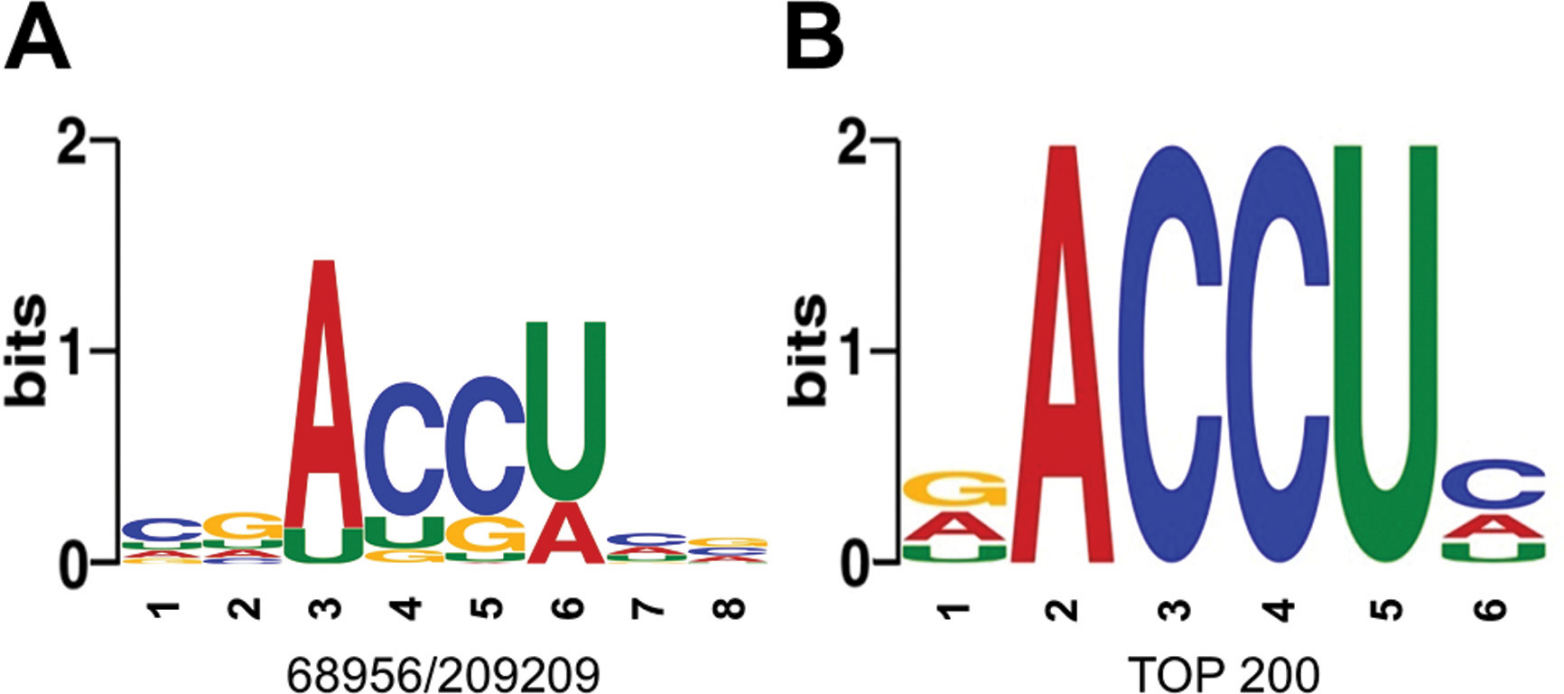
BsMiniIII cleavage site preference based on the NGS results. (**A**) The sequence motif obtained with DREME based on counting all cleavage sites. The numbers below the sequence motif indicate the fraction of occurrence of the most common motif instance over the total number of input sequences. (**B**) The sequence motif obtained with MEME based on the 200 most frequently cleaved unique sites.

In order to integrate the data obtained from mapping the ends onto both strands of the substrate RNA, the number of ends mapped on the bottom strand at a position X was added to the number of ends mapped on the top strand at a position X′+1. This procedure took into account the known geometry of the ends generated by the BsMiniIII cleavage (Figure 2E). We assumed that the minimal evidence for dsRNA cleavage by BsMiniIII was when the number of mapped reads was ≥1 for both positions X and X′+1. In order to identify the preferred cleavage sites, all reads that met the above condition were used as sequence motif instances, and their contribution to the final consensus calculation was weighted according to the number of counts of the ends mapped to the given site. Using DREME, we obtained a motif ACC^∧^U, where ^∧^ indicates the scissile phosphodiester bond (Figure 3A). If only 200 of the most frequent, unique sequence motifs were used for the consensus generation with MEME, essentially the same common motif was obtained (Figure 3B). Additionally, we performed the same analysis for products generated after 15 min of limited cleavage of the Φ6 genome. Despite a slightly higher level of noise, we obtained a similar consensus sequence ACC^∧^U, as in the case of results for the 10 min-limited cleavage (data not shown). Interestingly, the natural substrate of BsMiniIII, the pre-23S rRNA in *B. subtilis*, contains the ACC sequence in the cleavage site (17). Thus, the *in vitro* specificity of BsMiniIII is similar to that observed *in vivo* for the substrate that exhibits a partially double-stranded structure.

### Kinetic analysis of BsMiniIII cleavage

**Figure 4.**
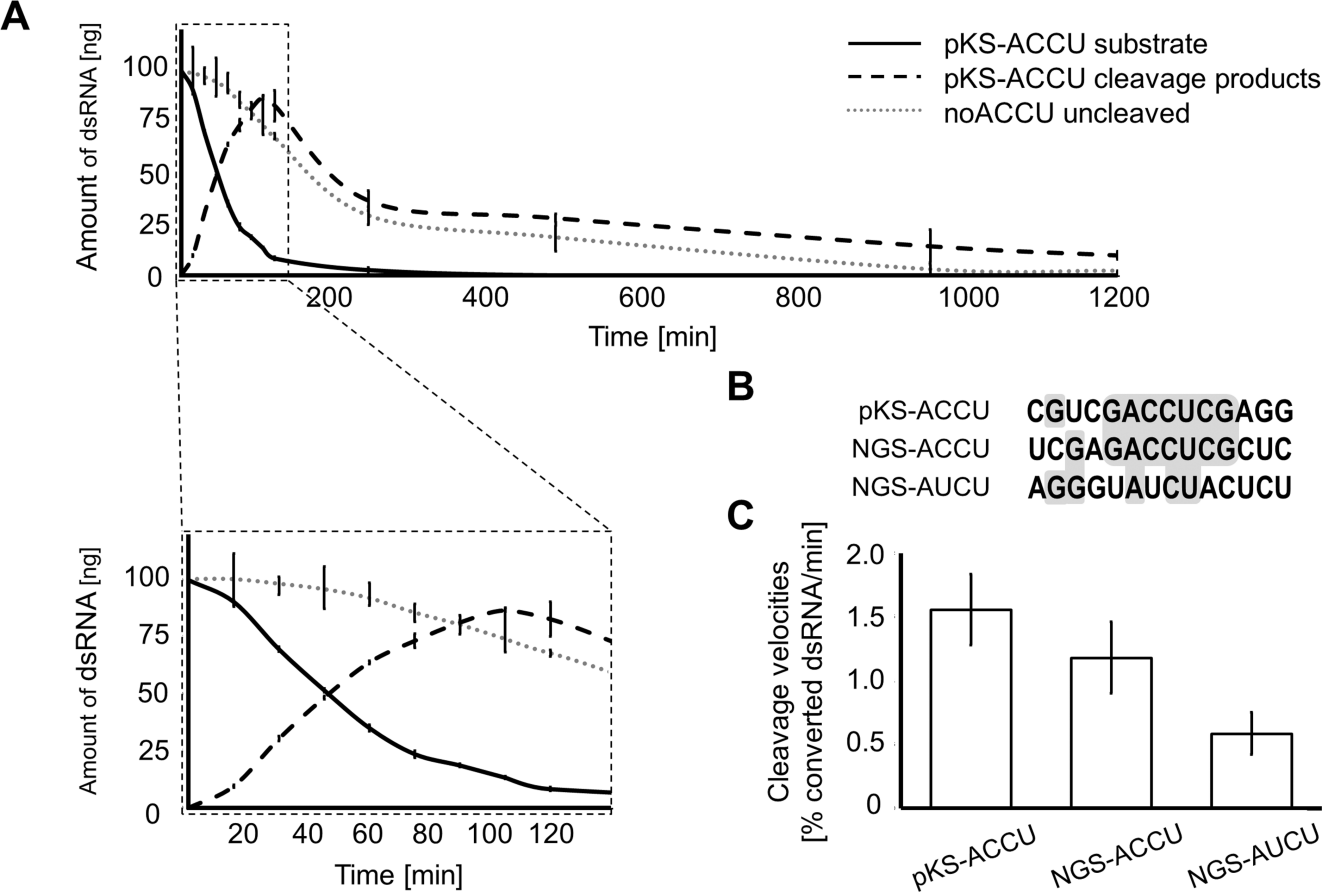
Cleavage of the selected substrate sequences by BsMiniIII. (**A**) A cleavage kinetics for two substrates with and without the ACCU sequence (pKS-ACCU and noACCU, respectively). The inset shows an expanded view of the progress of the initial reaction. Ten microliter aliquots containing 3.1 μg of BsMiniIII (8.3 μM) and 100-ng dsRNA (0.05 μM) were taken at timed intervals. Samples were resolved on native polyacrylamide gel, stained with ethium bromide and visualized using UV light. The amount of uncleaved substrate and generated products was measured densitometrically. (**B**) Sequence alignment of cleavage sites in pKS-ACCU, NGS-ACCU and NGS-AUCU substrates. Nucleotides identical in two or three sequences were highlighted in gray. (**C**) Initial cleavage rates of three substrates. Reactions were carried out in a manner similar to panel A and terminated after 15, 30 and 45 min. The percent of converted dsRNA/min was measured densitometrically. Cleavage velocities were normalized for the enzymes concentrations.

To gain additional insight into the cleavage preference of BsMiniIII, the single turnover cleavage kinetics (in the enzyme:substrate ratios from 2:1 up to 50:1) were studied for the substrate pKS-ACCU dsRNA containing a single instance of consensus sequence and for a 100 bp fragment of the Φ6 bacteriophage S segment that does not contain the central sequence ACCU (noACCU; Supplementary Figure S5). No substantial cleavage was detected within the latter fragment according to the Φ6 RNA cleavage assay, even at the highest enzyme to substrate ratio (Supplementary Figure S5C). In order to detect and measure the unspecific cleavage kinetics, we had to use in the analysis a very high enzyme to substrate ratio (100:1). Under such conditions, at the 105th min of reaction (Figure 4A), 88% of the pKS-ACCU dsRNA substrate was cleaved with concurrent accumulation of two defined products of the site-specific cleavage. At the same time, only 22% of noACCU dsRNA was cleaved, and in this case multiple products were created (observed as a smear in the gel electrophoresis). The extended cleavage of either substrate led to a complete degradation of RNA into a heterogeneous mixture of small fragments that did not form discrete bands in the gel electrophoresis. Our data indicate that the rates of unspecific degradation of noACCU and unspecific degradation of initial product of pKS-ACCU dsRNA-specific cleavage were similar and that the main difference in the degradation of both substrates was due to the specific cleavage of pKS-ACCU dsRNA by BsMiniIII.

We selected two sites from the Φ6 genome analysis with the highest sum of reads and with a ratio of reads from the two strands close to 1:1: UCGAG**ACC**^∧^**U**CGCUC (NGS-ACCU) and AGGGU**AUC**^∧^**U**ACUCU (NGS-AUCU) (^∧^ marks the cleavage site in the presented strand, where the four nucleotides of the motif around the cleavage site are in bold) to compare their cleavage rates with that of the pKS-ACCU dsRNA substrate. Alignment of the 14 nucleotides flanking these three cleavage sites showed that pKS-ACCU dsRNA and NGS-ACCU share a 7 bp fragment GACCUCG (Figure 4B). The NGS-ACUC substrate does not contain the ACCU sequence in the cleavage site (Figure 4B). The cleavage rates of dsRNA substrates containing these three sequences were measured (Figure 4C). The two substrates containing the ACCU sequence were cleaved with similar rates, whereas the cleavage rate of NGS-AUCU substrate was 62% lower, indicating that the ACCU motif is preferred, but is not absolutely essential for BsMiniIII cleavage.

### The crystal structure of BsMiniIII and the theoretical model of the enzyme–dsRNA complex

**Figure 5.**
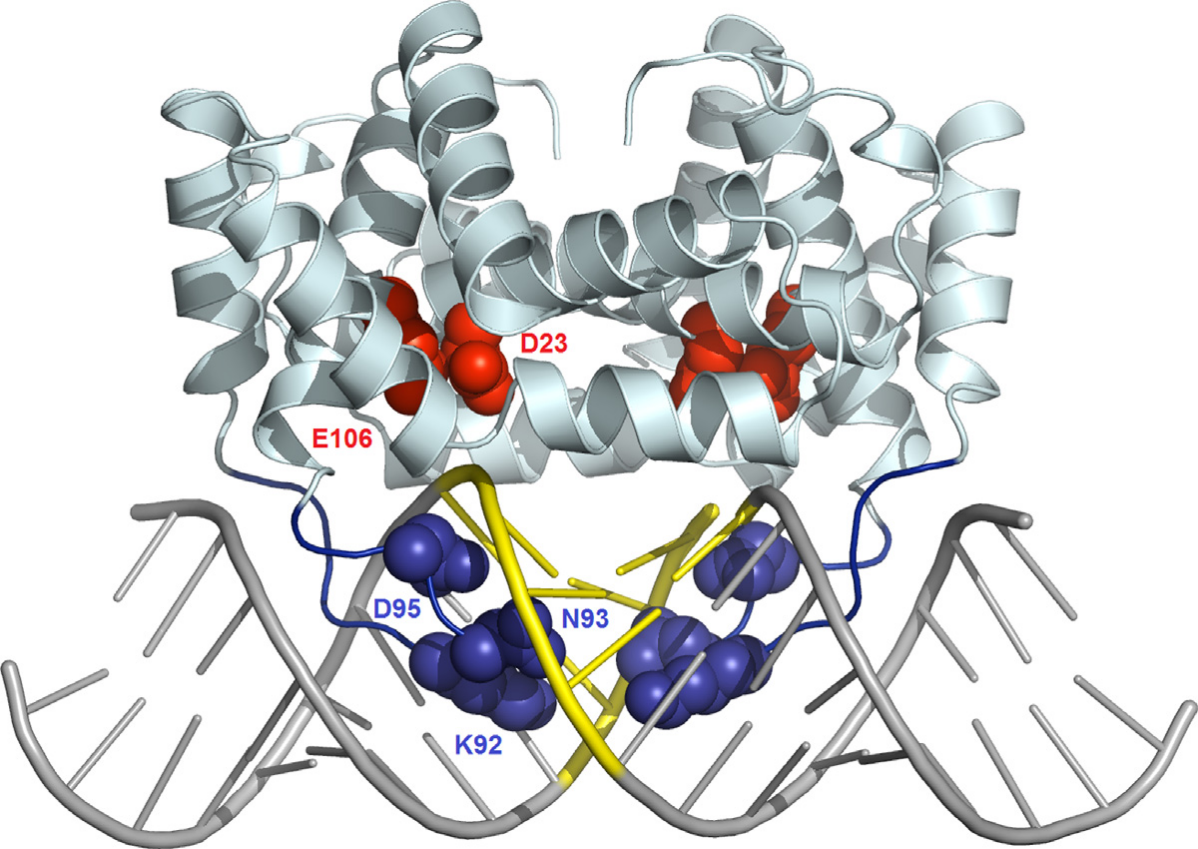
Model of BsMiniIII–dsRNA complex structure. The experimentally determined BsMiniIII structure is shown as a pale cartoon, and the modeled loop α5b-α6 is shown in blue. The homology-modeled dsRNA 20 mer is shown as gray sticks, with the BsMiniIII target site ACCU/AGGU in yellow. The side chains of putative catalytic residues conserved between MiniIII and RNase III are shown as red CPK balls. The residues predicted to interact with the target RNA sequence are shown as blue CPK balls. The coordinates of the model are available from ftp://genesilico.pl/iamb/models/RNases/eRNase_BsMiniIII/

Although structures of two Mini-III enzymes, from *B. cereus* and *Fusobacterium nucleatum*, are known (PDB IDs 1u61 and 2gsl), no structure of Mini-III RNase bound to dsRNA was reported. Since such structural information could provide crucial insight into the molecular details of the cleavage site selection, we attempted to crystallize a substitution variant (in one of the catalytic residues) of BsMiniIII with dsRNA. Until now, however, we managed to obtain only crystals of the free enzyme, not the complex with the substrate dsRNA. The structure was solved by molecular replacement and refined at 1.8-Å resolution to an R-free of 26.76% (Supplementary Table S4). Unfortunately, the loop α5b-α6, suggested to play a major role in contacting the RNA substrate (17), was not visible in the crystallographic model. To create a working structural model of BsMiniIII in complex with dsRNA, we used the crystal structure of *Aquifex aeolicus* RNase III complexed with dsRNA (12) as a template (PDB code: 2ez6). First, we superimposed the BsMiniIII structure onto the *A. aeolicus* RNase III complex structure and copied the coordinate of the RNA. We homology-modeled the BsMiniIII target dsRNA using ModeRNA (31) and finally modeled the missing loop, minimizing steric clashes between the protein and the RNA. The resulting model (Figure 5) suggested that the loop α5b-α6 in BsMiniIII, which is missing from the RNase III, can be easily placed in the major groove of the substrate, where it could make contacts with the target tetranucleotide sequence ACCU/AGGU. Therefore, we inferred that residues in this region may be responsible for sequence-specific recognition of the RNA substrate by BsMiniIII. Due to the lack of structural data of this region, we tested the importance of α5b-α6 loop residues for RNA recognition by deletion and site-directed mutagenesis.

### Interaction of a protein loop with the major groove of the dsRNA substrate

To validate the prediction of the importance of the loop α5b-α6 in BsMiniIII, variants with either 5- or 10-residue deletions (K86-D95 and P91-D95, respectively) were constructed. The binding affinity of these variants to the 30 bp dsRNA substrate was not affected (Supplementary Figure S6A), indicating that the loop α5b-α6 is not essential for dsRNA binding. However, both variants lost the RNase activity completely, suggesting that the presence of an intact loop α5b-α6 is indispensable for the cleavage of dsRNA by BsMiniIII (Supplementary Figure S6B).

**Figure 6.**
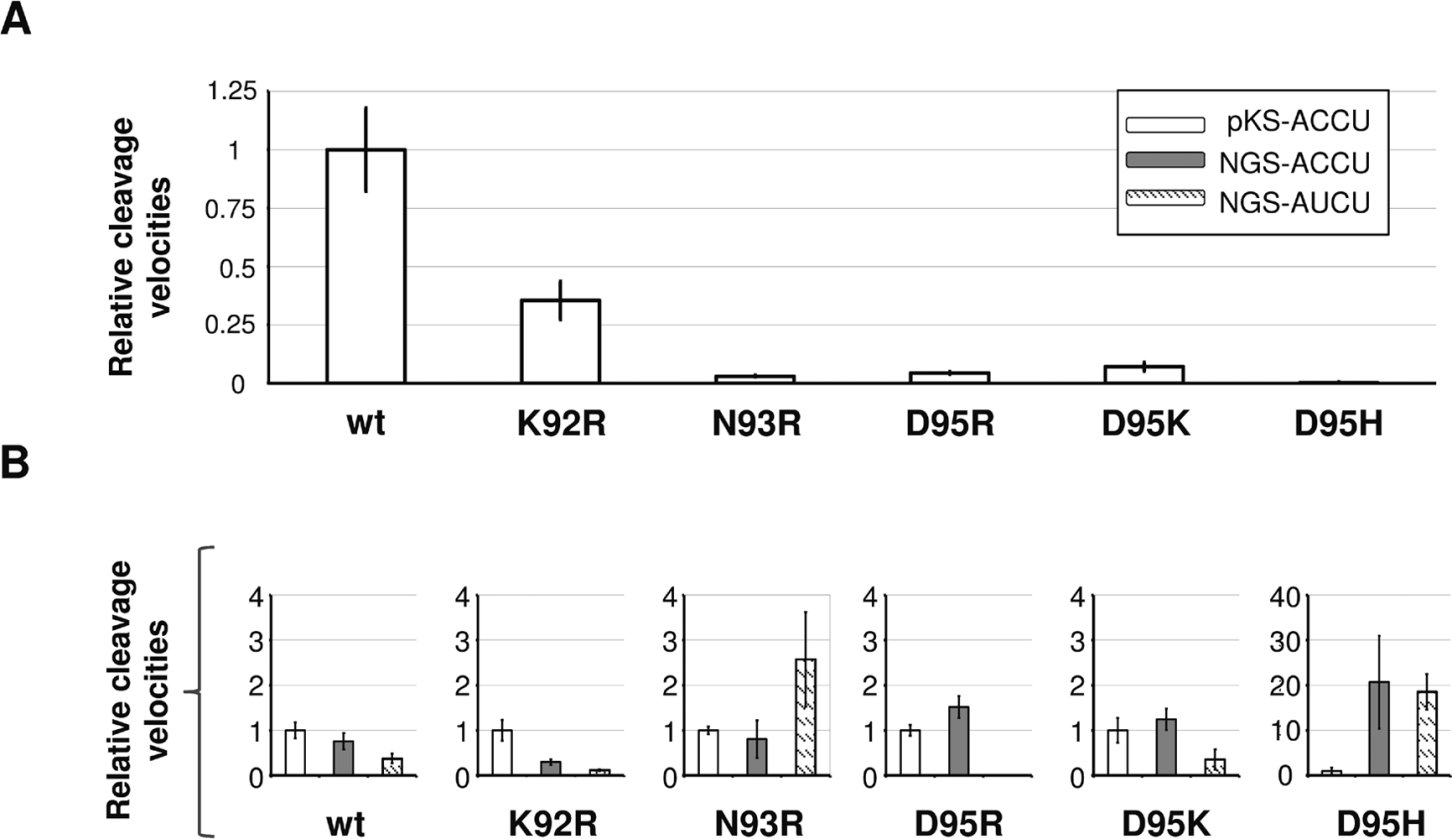
The effect of single substitution within the sequence loop α5b-α6 on BsMiniIII activity and its substrate preference. (**A**) The relative cleavage rates of pKS-ACCU dsRNA by wild-type BsMiniIII and its variants (see Supplementary Figure S7). The cleavage rates of the substitution variants were normalized to the cleavage rate of BsMiniIII, which was set to a value of 1. (**B**) The cleavage preferences of the wild-type BsMiniIII and its substitution variants. Normalization was made for each variant separately against the pKS-ACCU dsRNA cleavage rate.

In the initial step of the analysis, we introduced single alanine substitutions of the residues within the α5b-α6 loop, namely K80, R81, R83, N84, K86, S87, T89, K92, N93 and D95. Substitutions K80A, R81A, N84A, S87A and T89A had negligible effect on the cleavage activity (data not shown). Based on these results and the analysis of the model of BsMiniIII–dsRNA complex, we selected three amino acid residues that seemed to be most important for the target site selection (K92, N93 and D95) and tested the influence of their substitutions on the RNase activity. In total, 12 single substitution BsMiniIII variants (K92H, K92R, K92D, K92E, N93H, N93R, N93D, N93E, D95H, D95R, D95K and D95E) were tested. Seven out of these variants were inactive (products of cleavage were undetectable), whereas K92R, N93R, D95H, D95R and D95K retained detectable RNase activity (Supplementary Figure S7). In order to compare the properties of the enzyme variants, we measured the initial velocities of the pKS-ACCU dsRNA cleavage (Figure 6A). All of the tested substitution variants exhibited a marked decrease of the nucleolytic activity in comparison to the wild-type BsMiniIII. The cleavage rates of the NGS-ACCU and NGS-AUCU sequences were also measured. Similar to the results for the pKS-ACCU dsRNA substrate, the activity of the substitution variants was lower in comparison to the wild-type enzyme (Figure 6B). However, clear differences between the variants and the wild-type enzyme in the substrate preference were observed. For the wild-type enzyme, the cleavage preference can be summarized as pKS-ACCU>NGS-ACCU>NGS-AUCU. Only the variant K92R had a similar preference. None of the introduced substitutions affected the enzymes’ ability to bind dsRNA (data not shown) in agreement with our finding that even deletion of the entire loop does not interfere with dsRNA binding by BsMiniIII.

## DISCUSSION

Currently, the available data for members of the RNase III superfamily suggest that these enzymes recognize mainly the structure of their substrates, while the substrate sequence plays a minor role. Frequently, additional domains or associated proteins are involved in the recognition of natural RNA substrates. RNase III from *E. coli* has some sequence preference, but its consensus recognition site is very weakly defined and no particular bases are either absolutely required or absolutely excluded from any position (9). Until now, there were no reports for any member of the RNase III superfamily that exhibited substantial sequence preference that would make it suitable for precise fragmentation of long regular dsRNA molecules.

In the course of our analyses of BsMiniIII, a member of the Mini-III family, which was known to be involved in 23S pre-rRNA substrate processing, we discovered that it cleaves long dsRNA molecules into well-defined fragments without the need of any other protein cofactors. Initially, we serendipitously discovered a site in a dsRNA substrate (pKS-ACCU) that was specifically cleaved with high efficiency. Based on that finding, we used two complementary approaches to characterize the preferred BsMiniIII sequence. Our analysis of the cleavage of a set of dsRNA obtained using saturation mutagenesis of the pKS-ACCU substrate as well as the subsequent high-throughput analysis of products of cleavage of a dsRNA Φ6 phage genome resulted in very similar sequence motifs with a common core.

The results of high-throughput analyses were confirmed in a single-site cleavage assay also in the case of the NGS-ACUC substrate that undergoes efficient cleavage, despite its deviation from the consensus sequence. On the one hand, this serves as a validation of the reliability of high-throughput cleavage experiments, while on the other hand it demonstrates that there are some elements of the target site that are not well defined by the consensus sequence. The complicated pattern of bands, which is observed after limited cleavage of larger substrate such as the Φ6 genome (Figure 1A), is most probably the result of differences in the rates of the cleavage of multiple preferred sequences. During the RNA substrate digestion, a complex and dynamic mixture of partial cleavage products is generated. In the reaction conditions of high enzyme to substrate ratios at prolonged reaction times, the unspecific degradation of dsRNA without the preferred site was detectable and measurable. This characteristic of the activity of BsMiniIII seems to be similar to the activity of the canonical RNase III. Nevertheless, the kinetics of this step is very slow and the non-specific cleavage lags behind the initial, fast and specific cleavage of the preferred sequences.

BsMiniIII acts as a dimer, and thus it could be expected to prefer a symmetric site in dsRNA. However, we found that the enzyme strongly prefers ACCU/AGGU sequences with an asymmetric CC/GG dinucleotide pair in the center of the cleavage site. We found that palindromic substrates with either GC or CG sequence in the center are hardly cleaved or not at all.

Clearly, the structural data could help us elucidate the mechanism of the substrate selection by BsMiniIII. Unfortunately, despite extensive efforts, our attempts to crystallize BsMiniIII in complex with the dsRNA substrate failed, and we managed to obtain only a structure of the free enzyme. A model of the BsMiniIII–RNA complex structure led us to speculate that discrimination of the various dsRNA sequences by BsMiniIII reported in this work may be due to specific interactions of residues in loop α5b-α6 with the central bases of the dsRNA substrate. Accordingly, we demonstrated that the loop α5b-α6 in BsMiniIII is important for the dsRNase activitiy, even though it is completely dispensable for dsRNA binding. Further, several single residue substitutions in this loop either abolished the dsRNase activity of BsMiniIII or decreased it, as well as altered the cleavage sequence preference of the enzyme. Unfortunately, the current model is not accurate enough to warrant prediction of the protein–RNA contacts at the residue level and it cannot explain the asymmetry of the preferred target. Recognition of asymmetric sequences by homodimeric proteins is not unprecedented; e.g. it has been reported for the transcription factor GCN4 (32), a lambda phage repressor (33), a glucocorticoid receptor (34) and for the I-CeuI homing endonuclease (35). However, the elucidation of the details of the BsMiniIII-dsRNA sequence recognition must await the determination of a high-resolution complex structure.

Many molecular biology methods employ RNases. They are used for RNA footprinting and structure mapping (36), termination of the reverse transcription reaction (37), elimination of the unpaired RNA from the DNA sample (38) and in the RNase protection assay (39). What is still missing are enzymes that could be applied to more precise manipulations of RNA molecules. For instance, it is not always possible to generate by *in vitro* transcription an RNA molecule with a desired sequence on either the 5′ or the 3′ end. The availability of sequence-specific RNases in a molecular biology toolbox would extend our capacity to manipulate RNA *in vitro* and would facilitate the creation of ‘building blocks’ with a given RNA sequence and structure (40–42). It is important to point out that almost all native RNA molecules contain locally double-stranded regions. The applicability of the sequence-specific RNases is therefore not limited to RNAs containing dsRNA structures alone. In this context, we must emphasize also the fact that the sequence of the BsMiniIII cleavage site present in its native 23S pre-rRNA substrate is similar to the consensus sequence described in this work. Thus, BsMiniIII exhibits a clear sequence preference and we speculate that it can cleave specifically not only the element in 23S pre-rRNA or ideal dsRNA, but also other substrates, both *in vivo* and *in vitro*. It is noteworthy that *Pseudomonas syringae*, the host of dsRNA bacteriophage Φ6, does not encode a MiniIII RNase. It would be interesting to determine whether introduction of a functional MiniIII gene from another species into *Pseudomonas* may interfere with Φ6 replication *in vivo*.

Our demonstration of a sequence-specific cleavage of dsRNA by BsMiniIII puts it forward as a prototype of sequence-specific dsRNases and an attractive potential target for protein-engineering efforts. It will be interesting to determine if other members of the Mini-III family exhibit sequence specificity and if any of them prefer targets other than AGGU/ACCU.

## ACCESSION NUMBER

Atomic coordinates have been deposited in the Protein Data Bank (www.rcsb.org) under accession code 4OUN.

## SUPPLEMENTARY DATA

Supplementary Data are available at NAR Online.

SUPPLEMENTARY DATA
