# Supplementary material for: Sequence-specific cleavage of dsRNA by Mini-III RNase
Source: Nucleic Acids Res. 2015 Jan 29;43(5):2864–73. doi: 10.1093/nar/gkv009 (PMC4357697; doi:10.1093/nar/gkv009)
Supplement: SUPPLEMENTARY DATA [file supp_43_5_2864__index.html]

Sequence-specific cleavage of dsRNA by Mini-III RNase — Sequence-specific cleavage of dsRNA by Mini-III RNase — SUPPLEMENTARY DATA 

# Sequence-specific cleavage of dsRNA by Mini-III RNase

## SUPPLEMENTARY DATA

**Files in this Data Supplement:**

- SUPPLEMENTARY DATA
