# Supplementary material for: Sequence-specific cleavage of dsRNA by Mini-III RNase
Source: Nucleic Acids Res. 2015 Jan 29;43(5):2864–73. doi: 10.1093/nar/gkv009 (PMC4357697; doi:10.1093/nar/gkv009)
Supplement: SUPPLEMENTARY DATA [file supp_gkv009_nar-02570-h-2014-File008.docx]

**Supplementary materials:**

**Supplementary material and methods:**

## Filter binding assay:

The nitrocelullose filters were soaked for 10 min in binding buffer (10 mM Tris-HCl pH 7.5, 5 mM NaCl, 1 mM CaCl_2_) at room temperature. The binding reactions were carried out for 1 hour at room temperature in 50 μl total volume with 0,01 µM ^32^P end labeled 30 bp dsRNA and various concentrations of enzymes (from 0,1µM to 4µM). The filters were placed on a 96-well filter manifold (Biorad) and vacuum was applied to remove the excess moisture. The binding reactions were then filtered followed by washing with 150 µl of the binding buffer. The filters were dried and the radioactivity was visualized by autoradiography on the Storm scanner (GE Healthcare) from Storage Phosphor Screen (GE Healthcare). The intensities of dots were measured by ImageQuant TL (GE Healthcare). The dissociation constants were calculated by Sigma Plot (Systat Software Inc.) using the single-site specific binding model.

**Mass spectrometry:**

To analyze the potential contamination of our BsMiniIII preparation with *E. coli* RNase III, an aliquot of the protein sample was analyzed in MALDI-MS. The protein sample (1 μg) was desalted on C-18 tip (Supelco) according to the manufacturer instructions. The eluted protein was mixed in 1:1 proportion with SDHB matrix (Sigma) dissolved at 50 mg/ml in 50% acetonitrile in 0.1% trifluoroacetic acid and spotted on ground steel target plate. The MS spectra were collected in ultrafleXtreem spectrometer (Bruker) and calibrated using Protein Calibration Standard II mixture (Bruker).

Another aliquot of the protein preparation was digested with trypsin (Thermo) according to protocol recommended by the supplier, desalted on C-18 tip (Supelco) and applied to LC‑MS-MS analysis using Easy Nano-LC chromatography system (Proxeon) connected with amaZon mass spectrometer (Bruker). The elution was done by 30 minutes 0-100% B linear gradient (buffer A: 0.1% formic acid in water; buffer B: 0.1% formic acid in acetonitrile). Mass spectra were analyzed on local Mascot server using database containing *E. coli* BL21(DE3) proteome plus BsMiniIII sequence.

**Supplementary Figures:**


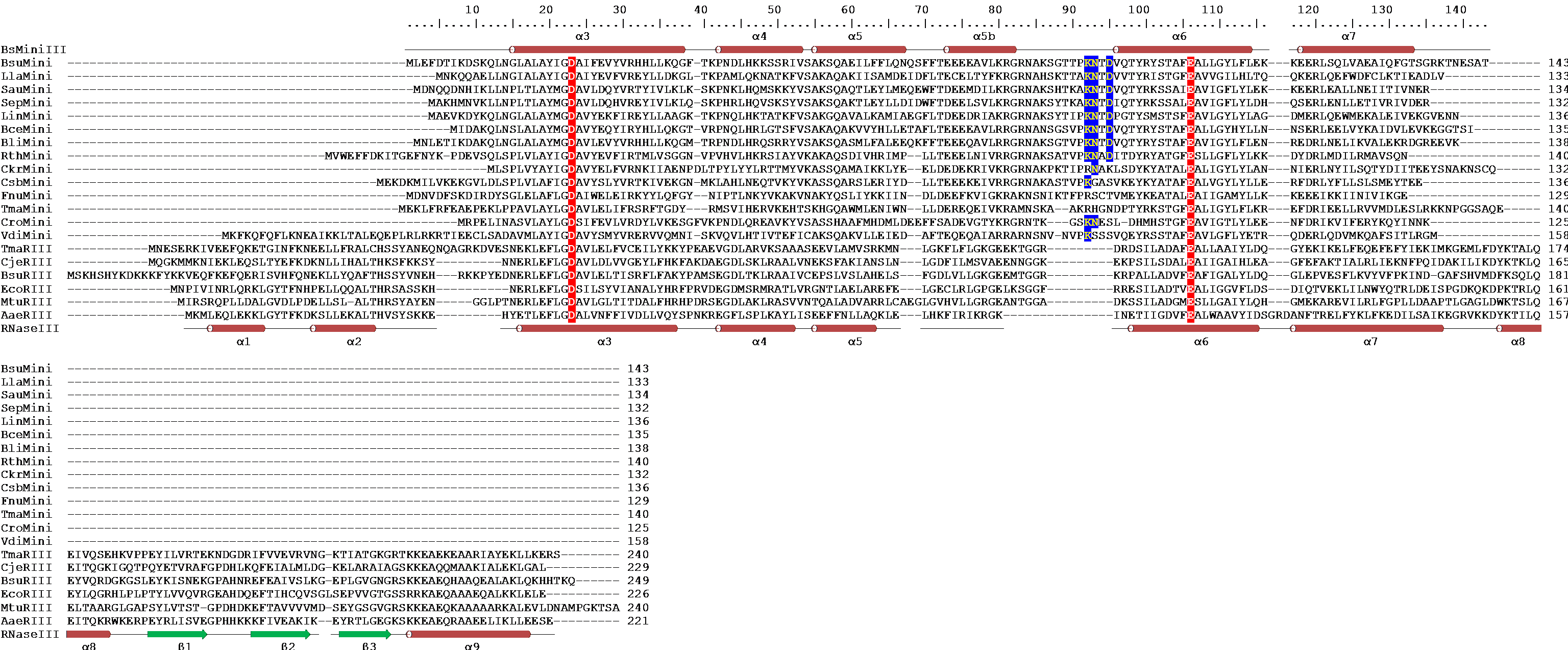


**Figure S1. Alignment of Mini-III and RNase III protein sequences from selected organisms** (Aae, *Aquifex aeolicus*; Bsu, *Bacillus subtilis*; Bce, *Bacillus cereus*; Eco, *Escherichia coli*; Tma, *Thermotoga maritima*; Cje, *Campylobacter jejuni*; Mtu, *Mycobacterium tuberculosis*; Bli, *Bacillus licheniformis*; Lin, *Lsteria innocua*; Sau, *Staphylococcus aureus*; Lla, *Lactococcus lactis*; Fnu, *Fusobacterium nucleatum*; Tma, *Thermotoga maritima*; Vdi, *Veillonella dispar*; Csb, *Caldanaerobacter subterraneus*; Sep, *Staphylococcus epidermidis*; Ckr, *Caldicellulosiruptor kristjanssonii*; Rth, *Ruminiclostridium thermocellum*; Cro, *Clostridium ramosum*). The secondary structures of Mini-III from *B. subtilis* (determined in this work) and RNaseIII from A. aeolicus (derived from PDB: 2ez6) are shown on the bottom and top respectively (cylinders represent α-helices; arrows represent β-strands). Conserved residues of the active site are indicated in red (D23 and E 106 in BsMiniIII), and residues studied in detail by mutagenesis in BsMiniIII (K92, N93, and D95) and their counterparts in other MiniIII sequences are indicated in blue.

.


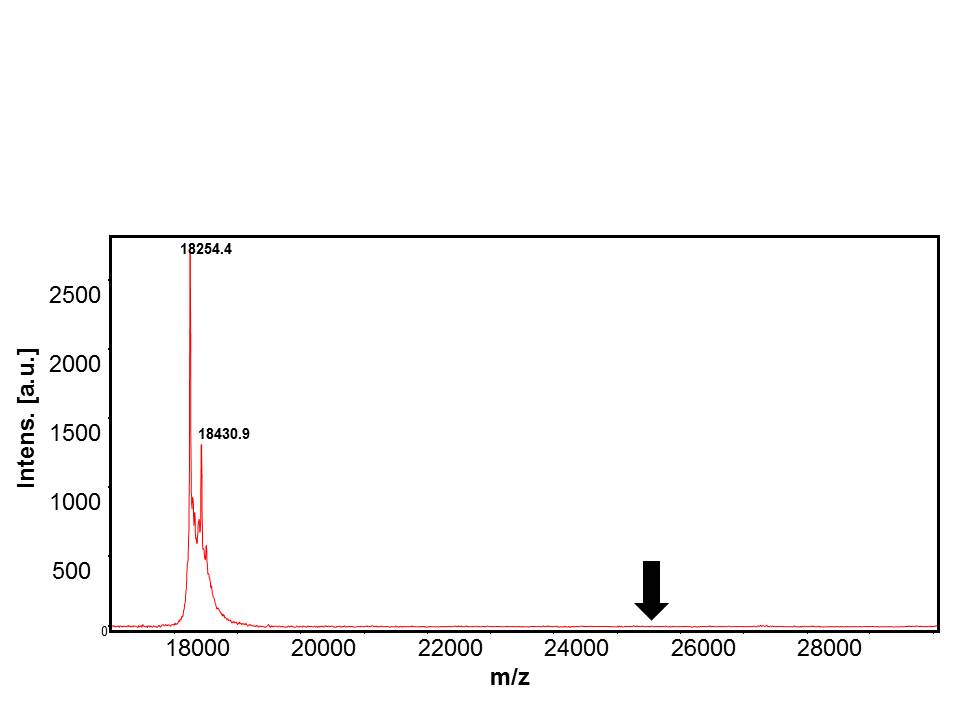


**Figure S2.** **Mass spectrometry analysis of the BsMiniIII sample.** The MALDI MS spectrum of the desalted BsMiniIII sample. m/z of two major ions are shown. Ion of m/z = 18,430 probably represents full length BsMiniIII with N-terminal N-formylmethionine (calculated m/z = 18,382). The ion of m/z = 18,254 is most probably BsMiniIII with N-formylmethionie removed (calculated m/z = 18,251). Black arrow indicates the position where +1 ion of *E.coli* RNaseIII should appear (calculated m/z = 25,551).

**
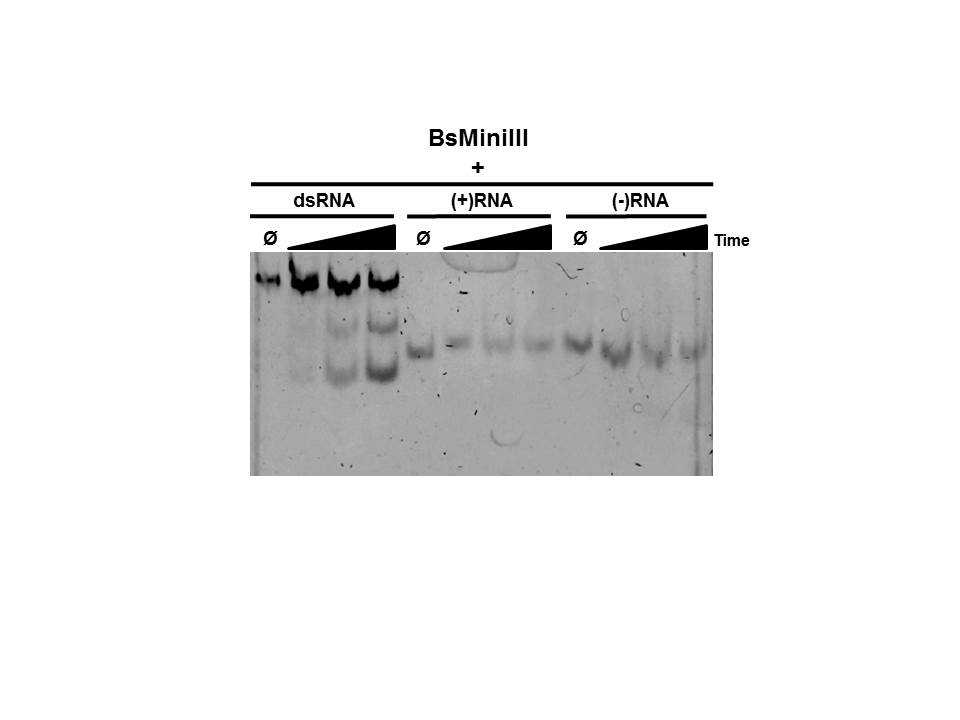
**

**Figure S3. BsMiniIII cleavage reaction of 30 nt dsRNA with ACCU sequence and each strand separately.** The incubation times were: 60, 120 and 180 min. The reactions were carried out at 37 °C. 0.17 μM RNA was incubated with 1.7 μM BsMiniIII.

**
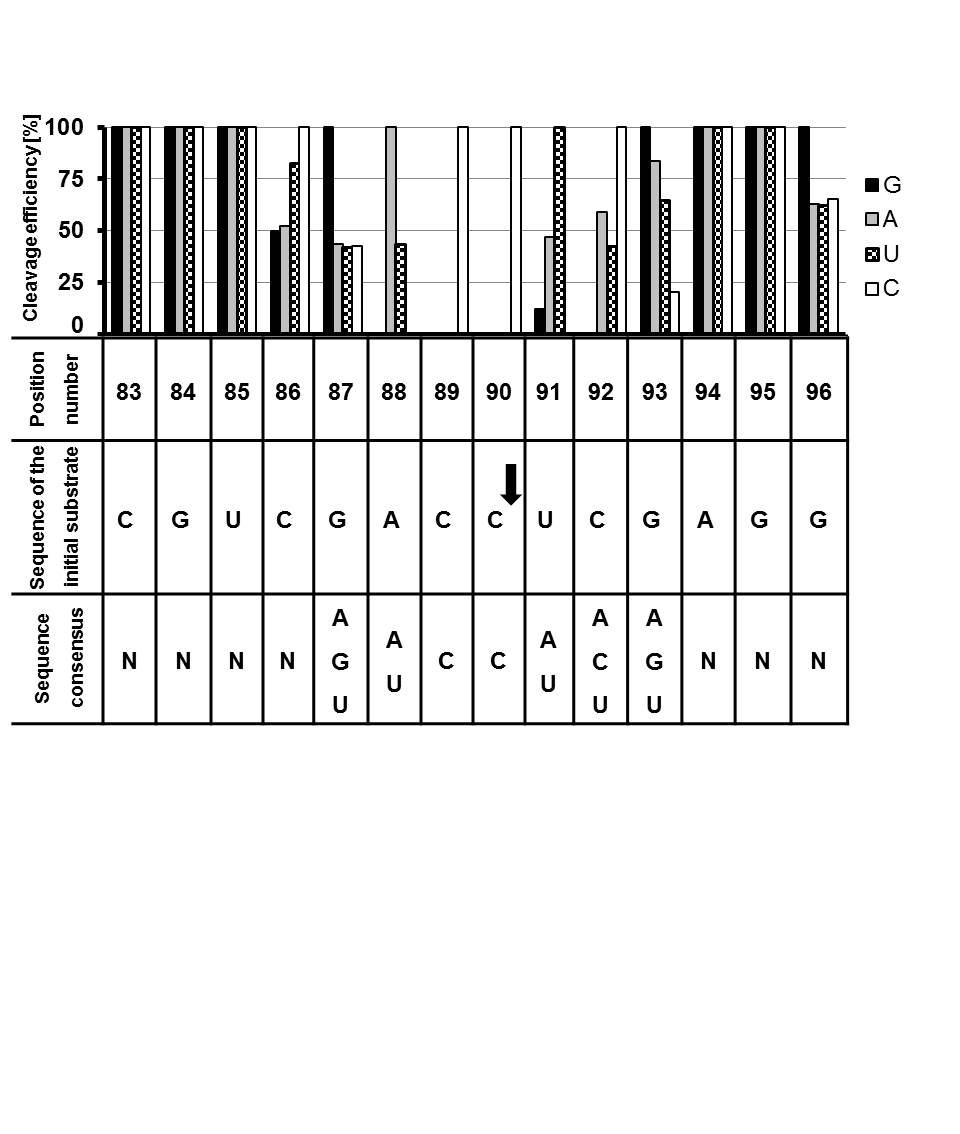
**

**Figure S4.** **Influence of point substitutions in the sequence flanking cleavage site of pKS‑ACCU on the efficiency of cleavage by BsMiniIII.** The cleavage of the single substitution variants of pKS-ACCU dsRNA substrate by BsMiniIII. The reactions were carried out for 1 h at 37°C at 35:1 enzyme : substrate molar ratio. The amount of the uncleaved substrate and the generated products was measured by densitometry. Cleavage efficiencies are normalized to efficiency for the original pKS-ACCU substrate. The sequence consensus represents all of the sequences that were cleaved with the efficiency greater than 25%. The arrow indicates the cleavage site.


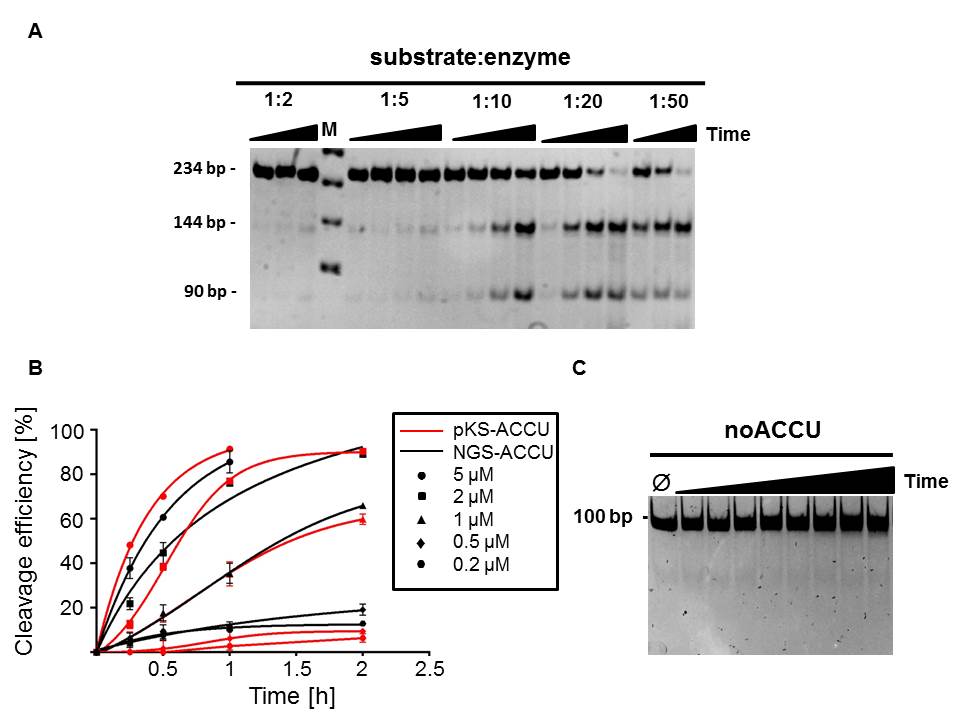


**Figure S5. Kinetics of the specific cleavage of BsMiniIII.** A. The cleavage reactions of pKS-ACCU substrate with different enzyme:substrate ratios (indicated above the gel). The substrate concentration was 0.11 µM. The enzyme concentrations were 0.2 µM, 0.5 µM, 1 µM, 2 µM and 5 µM. Aliquots were taken at 15, 30, 60 and 120 minutes. Last and first time point aliquots are not shown for 1:50 and 1:2 ratio respectively. B. The results of the densitometry analysis of three independent cleavage experiments using two substrates pKS-ACCU and NGS-ACCU. The substrate concentration was 20 ng/µl (0.11 µM for pKS-ACCU and 0.08 µM for NGS-ACCU). **C.** Cleavage reaction of noACCU substrate with substrate:enzyme molar ratio of 150:1 (8.3 µM BsMiniIII). Aliquots were taken at 15, 30, 45, 60, 75, 90, 105 and 120 min. The samples were resolved on a native polyacrylamide gel.


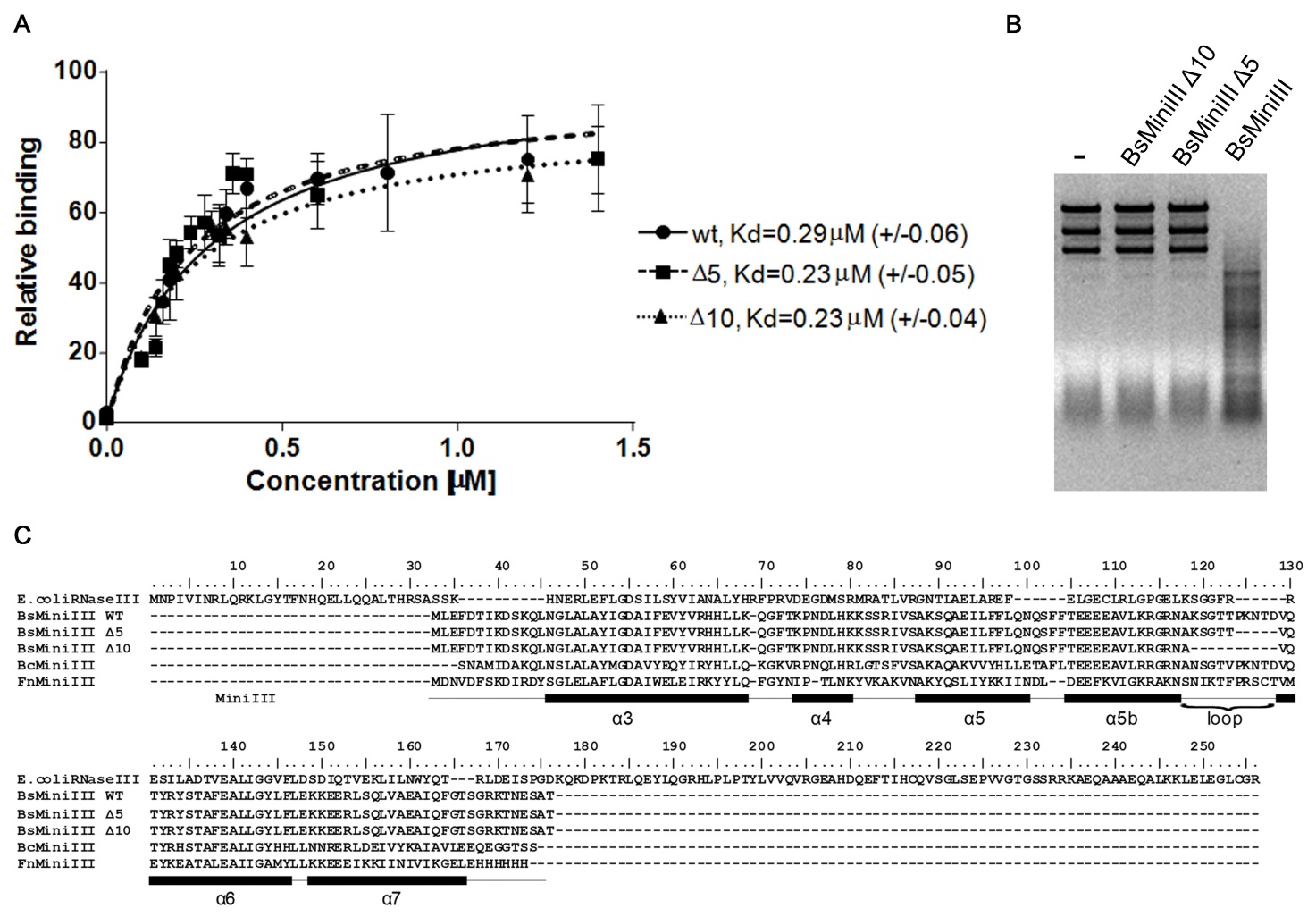


**Figure S6.** A. Binding affinity of BsMiniIII and the two deletion variants to the 30 bp dsRNA substrate measured in a filter binding assay. B. The cleavage of the Φ6 dsRNA substrate by BsMiniIII and two deletion variants (BsMiniIII Δ5 and BsMiniIII Δ10).


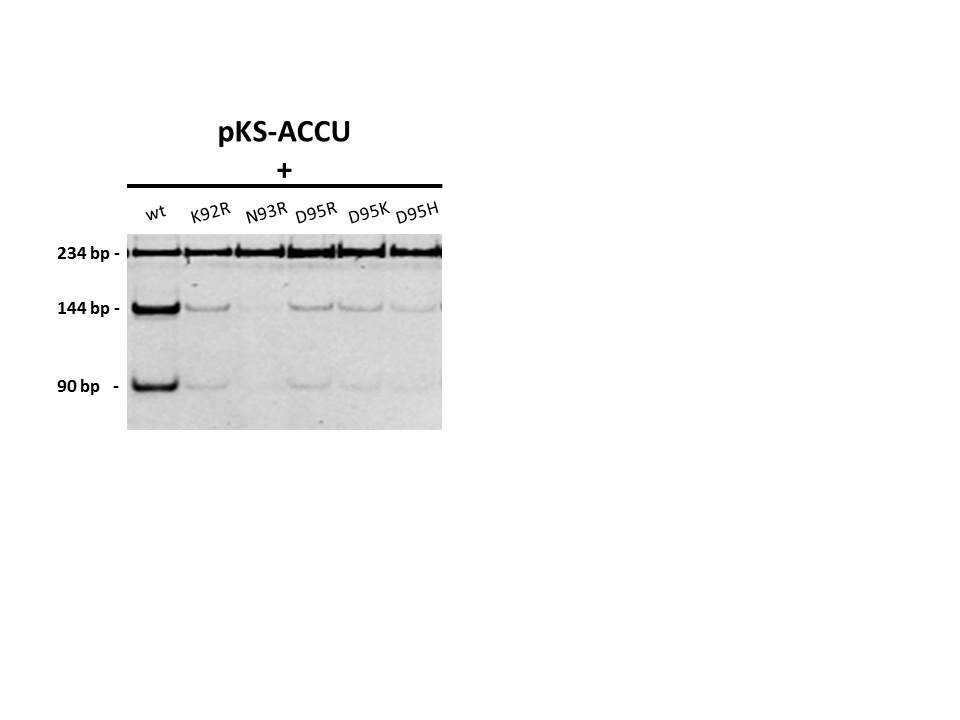


**Figure S7. The effect of single amino acid substitutions within loop α5b-α6 on BsMiniIII activity.** Cleavage reactions were carried out for 45 min at 37°C. For each 10 μl of reaction volume 100 ng dsRNA (0.05 µM) and 3.1 µg of wt BsMiniIII (8.3 μM) or 5 µg (13.4 μM) of BsMiniIII substitution variant was used. Samples were resolved on a native polyacrylamide gel.

**Supplementary Tables:**

**Table S1.** Primers

| Primer name | Primer sequence |
| --- | --- |
| BSUf | ATCCATGGTTGAATTTGATACGATAAAAGATTC |
| BSUr | TACTCGAGTGTTGCTGACTCATTTG |
| BSUΔf | GTTCAGACGTACCGCTACAG |
| BSU5r | TGTTGTCCCTGACTTGGC |
| BSU10r | GGCATTTCTGCCTCTTTTCAGCAC |
| KSf | ATCCCCCGGGCTGCAGGAATTC |
| KSr | CCCTATAGTGAGTCGTATTACGCGCG |
| 30f | CGATACCGTCGACCTCGAGGGGGGGCCCGG |
| 30r | CCGGGCCCCCCCTCGAGGTCGACGGTATCG |
| UniShPreA | AGATCGGAAGAGCGTCGTGTAGGGAAAGAGTGTAGA |
| UniShRT | TCTACACTCTTTCCCTACACGAC |
| PreA3Univ | GATCGGAAGAGCACACGTCTGAACTCCAGTCAC |
| noACCUf | TAATACGACTCACTATAGGGCCTCTCTCTCTGGCCACGATC |
| noACCUr | GGAAAAAAATGCCCTGTACAGCAGGCATAAG |
| NGS-AUCUf | TAATACGACTCACTATAGGGTACCGCGATCAACACTGTCGTC |
| NGS-AUCUr | GGAAAAAAACGAATCAGGACGTCTGGACG |
| NGS-ACCUf | TAATACGACTCACTATAGGGCTCCTATCATGGCCGTTGC |
| NGS-ACCUr | GGAAAAAAACTTCGAGATCAGGGTTGGACG |
| BsK86Nf | ACTCAGGGACAACACCTAAAAATACAG |
| BsK92Rf | CGAAATACAGATGTTCAGACGTACC |
| BsK92Hf | CATAATACAGATGTTCAGACGTACC |
| BsK92Df | GATAATACAGATGTTCAGACGTACC |
| BsK92Ef | ACAAATACAGATGTTCAGACGTACC |
| BsN93Rf | CGAACAGATGTTCAGACGTACC |
| BsN93Hf | CATACAGATGTTCAGACGTACC |
| BsN93Df | GATACAGATGTTCAGACGTACC |
| BsN93Ef | ACAACAGATGTTCAGACGTACC |
| BsD95Hf | CATGTTCAGACGTACCGCTACAGTACAG |
| BsD95Kf | AAGGTTCAGACGTACCGCTACAGTACAG |
| BsD95Rf | CGTGTTCAGACGTACCGCTACAGTACAG |
| BsD95Ef | ACAGTTCAGACGTACCGCTACAGTACAG |
| BsK86r | TGGCATTTCTGCCTCTTTTCAGC |
| BsK92r | AGGTGTTGTCCCTGACTTG |
| BsN93r | TTTAGGTGTTGTCCCTGACTTG |
| BsD95r | `TGTATTTTTAGGTGTTGTCCCTGACTTG |
| BsE105Qf | GGCGCTTCTGGGCTACCTTTTTC |
| BsE105r | TGAAATGCTGTACTGTAGCGGTAC |

**Table S2.** Mascot identification of potential contaminants in the BsMiniIII preparation.

| Accession | Protein | Number of identified peptides | Molecular mass (kDa) | Isoelectric point | Mascote score |
| --- | --- | --- | --- | --- | --- |
| MINIIII_BSU | Mini III | 5 | 18.4 | 9.5 | 616.4 |
| C6EEL6_ECOBD | Acid resistance protein | 2 | 83.4 | 6.3 | 28.3 |
| C6EFY1_ECOBD | UPF0259 membrane protein YciC | 1 | 26.4 | 10.6 | 39.5 |
| C5W9Z4_ECOBD | Phosphorylase | 1 | 90.5 | 7.3 | 35.1 |
| C6EEH1_ECOBD | Efflux transporter, RND family, MFP subunit | 1 | 41.2 | 5.6 | 34.8 |
| C6EEZ0_ECOBD | L-rhamnose-proton symporter | 1 | 37.3 | 10.1 | 34.7 |
| C6E9V0_ECOBD | Outer membrane protein | 1 | 18.6 | 4.6 | 33.4 |
| C6EBI8_ECOBD | Phosphopentomutase | 1 | 44.3 | 5.0 | 33.1 |
| C6EK51_ECOBD | Leucine--tRNA ligase | 1 | 97.2 | 5.0 | 33.1 |
| C6EIB1_ECOBD | L-allo-threonine aldolase, PLP-dependent | 1 | 36.5 | 5.8 | 33.0 |

The search was run against proteome of *E. coli* BL21(DE3) supplemented with the sequence of MiniIII from *B. subtilis*. Ten best scores are presented although only the first one passes the identification threshold. *E. coli* RNase III is not listed among all 51 identifications which were considered by the Mascot but rejected because of the low scores.

**Table S3.** Primers used for mutagenesis of the preferred target sequence.

| Library number | Nucleotide position substitution number | Forward primer | Reverse primer |
| --- | --- | --- | --- |
| 1 | 83 | Subf  CTCGAGGGGGGGCCCGGTA | Sub83r  GTCGACHGTATCGATAAGCTTG |
| 2 | 84 |  | Sub84r  GTCGADGGTATCGATAAGCTTG |
| 3 | 85 |  | Sub85r  GTCGBCGGTATCGATAAGCTTG |
| 4 | 86 |  | Sub86r  GTCHACGGTATCGATAAGCTTG |
| 5 | 87 |  | Sub87r  GTDGACGGTATCGATAAGCTTG |
| 6 | 88 |  | Sub88r  GVCGACGGTATCGATAAGCTTG |
| 7 | 89 |  | Sub89r  HTCGACGGTATCGATAAGCTTG |
| 8 | 90 | Sub90f  DTCGAGGGGGGGCCCGGTA | Subr  GTCGACGGTATCGATAAGCTTG |
| 9 | 91 | Sub91f  CVCGAGGGGGGGCCCGGTA |  |
| 10 | 92 | Sub92f  CTDGAGGGGGGGCCCGGTA |  |
| 11 | 93 | Sub93f  CTCHAGGGGGGGCCCGGTA |  |
| 12 | 94 | Sub94f  CTCGBGGGGGGGCCCGGTA |  |
| 13 | 95 | Sub95f  CTCGAHGGGGGGCCCGGTA |  |
| 14 | 96 | Sub96f  CTCGAGHGGGGGCCCGGTA |  |

**Table S4.** Crystallographic data collection and refinement statistics.

| Space group | C 2 2 21 |
| --- | --- |
| Unit cell dimensions | a =41.092Å  b =62.028Å  c =89.388Å  α = 90.00°  β = 90.00°  γ = 90.00° |
| No. of monomers / asymmetric unit | 1 |
| Independent reflections (all/test) | 10939 / 547 |
| Resolution range [Å] | 44.69 - 1.8 |
| Completeness [%] (last shell) | 99.84 (99.0) |
| I / σ (last shell) | 17.24 (2.11) |
| B–factor from Wilson plot [Å2] | 32.90 |
| R-factor [%] | 20.60 |
| R-free [%] | 26.76 |
| rmsd bond distance [Å] | 0.015 |
| rmsd bond angles [deg.] | 1.54 |
| Average B [Å2] | 42.10 |
| Ramachandran favored [%] | 97.0 |
| Ramachandran outliers [%] | 0.0 |
| Clashscore | 6.94 |
